# Comparison of the Efficacy of Fluorometholone With and Without Benzalkonium Chloride in Ocular Surface Disease

**DOI:** 10.1097/ICO.0000000000000695

**Published:** 2015-11-30

**Authors:** Yeoun-Hee Kim, Jae-Chang Jung, Soon-Young Jung, Sung Yu, Kyoo Won Lee, Young Jeung Park

**Affiliations:** * Cheil Eye Research Institute, Cheil Eye Hospital, Daegu, South Korea; and; † Developmental Biology Laboratory, Department of Biology, College of Natural Sciences, Kyungpook National University, Daegu, South Korea.

**Keywords:** fluorometholone, dry eye, preservative, cytotoxicity, TNF-α, IL-6, HLA-DR

## Abstract

**Purpose:**

The purpose of this study was to compare the cytotoxicity and antiinflammatory effect of preserved and unpreserved 0.1% fluorometholone (FML).

**Methods:**

Drug-induced morphological changes and cytotoxicity were examined in human corneal epithelial cells. Dry eye was induced in mice by treatment with 0.2% benzalkonium chloride (BAC) for the first 2 weeks, and then, the eyes (4 groups; Normal saline, BAC, preserved FML, and unpreserved FML) were treated thrice daily with each formulation for the next 2 weeks. Corneal tissues were embedded in paraffin and stained with hematoxylin and eosin for histopathological examination. Immunofluorescence staining was performed for tumor necrosis factor-α, interleukin-6, and human leukocyte antigen–DR. Terminal deoxynucleotidyl transferase dUTP nick end labeling assay was performed to evaluate drug-induced cytotoxicity.

**Results:**

BAC and preserved FML caused cell shrinkage and detachment from the plate in a dose-dependent manner, and cell viability decreased significantly. However, cytotoxicity was reduced on treatment with unpreserved FML. Hematoxylin-eosin staining revealed surface desquamation, irregular surface, loss of cell borders, and stromal shrinkage in the group treated with BAC. On BAC exposure, tumor necrosis factor-α, interleukin-6, and human leukocyte antigen–DR were strongly detected, and cytotoxicity was markedly increased, as evidenced by a positive result in the terminal deoxynucleotidyl transferase dUTP nick end labeling assay. Ocular surface damage and inflammation were slightly reduced on treatment with preserved FML. In comparison, unpreserved FML did not induce morphological changes; moreover, decreased cell cytotoxicity and ocular surface inflammation were observed.

**Conclusions:**

The cytotoxicity of antiinflammatory eye drops evaluated in this study was induced by the preservative BAC. Accordingly, unpreserved FML is more effective than preserved eye drops in decreasing ocular inflammation.

Many topical eye drop solutions used to treat corneal diseases contain preservatives to prevent contamination by microorganisms. The most commonly used preservative in ophthalmic preparations is benzalkonium chloride (BAC).^1^ As a soluble antimicrobial agent and a cationic surfactant, BAC enhances the penetration of active compounds. A recent study has revealed that some of the side effects of eye drops are caused by preservatives, such as BAC.^2^ Cytotoxicity of BAC on the ocular surface has been examined using in vitro cell culture systems, such as human corneal cell lines,^3^–^5^ human conjunctival cell lines,^3^,^4^,^6^,^7^ a 3-dimensional model of human corneal epithelium,^8^ and rabbit corneal cell lines.^6^,^9^ The ocular surface toxicity of BAC has also been investigated in several in vivo experiments by applying BAC to the ocular surface of mice,^10^,^11^ rats,^12^ and rabbits.^13^,^14^ Because of its toxicity, the use of BAC in eye drops has been criticized in recent years. BAC may induce ocular surface changes, causing ocular discomfort, tear film instability, loss of goblet cells, inflammation, conjunctival squamous metaplasia, epithelial apoptosis, subconjunctival fibrosis, and the potential risk of failure during future glaucoma surgery.^15^ Several studies have revealed that BAC is detrimental to ocular surface tissues and cells. Therefore, efforts are being made to reduce the adverse effects of BAC, and novel BAC-free ophthalmic formulations have been developed over the last several years.^16^,^17^ In addition, new preservatives with a wide range of activity and optimal safety profiles have been introduced.^18^

Dry eye syndrome is one of the most common multifactorial ocular surface diseases, affecting millions of people worldwide, and resulting in ocular dryness, discomfort, visual disturbance, and instability of tear film.^19^ Ocular surface inflammation may play a critical role in the pathogenesis of dry eye syndrome. Disease or dysfunction of tear secretory glands leads to changes in tear composition, such as hyperosmolarity, which stimulates the production of inflammatory mediators on the ocular surface.^19^,^20^ In turn, inflammation may cause dysfunction or loss of cells responsible for tear secretion or retention.^21^ Therefore, antiinflammatory therapy has been adopted as a common therapeutic strategy for the treatment of dry eye syndrome.

Commonly used antiinflammatory eye drops, such as topical corticosteroids and cyclosporine A, are available for the treatment of dry eye syndrome.^22^,^23^ Therefore, it is desirable to identify and evaluate suitable ophthalmic antiinflammatory agents. Fluorometholone (9a-fluoro-11b,17a-dihydroxy-6a-methylpregna-1,4-diene-3,20-dione; FML) is a corticosteroid used for its glucocorticoid activity. This drug is formulated in the form of an ophthalmic solution for the treatment of allergic conjunctivitis and vernal keratoconjunctivitis.^24^ In particular, FML is an effective antiinflammatory drug widely used to control eye inflammation.^25^

This study was designed to evaluate the cytotoxicity and antiinflammatory effect of BAC by comparing BAC-containing FML with BAC-free FML, both of which are commercially available, in human corneal epithelial cells (HCECs) and a dry eye mouse model.

## MATERIALS AND METHODS

### Materials

BAC was purchased from Sigma (Sigma-Aldrich, St. Louis, MO). Preserved (0.01% BAC) 0.1% FML was purchased from Alcon (Flucon; Alcon Laboratories, Fort Worth, TX), and unpreserved 0.1% FML, FML powder, and original solvent were obtained from Hanlim (Fumelone eye drops 0.6 mL; Hanlim Pharm. Co., Seoul, South Korea). Antitumor necrosis factor (TNF)-α, antiinterleukin (IL)-6, and antihuman leukocyte antigen (HLA)-DR antibodies were purchased from Abcam (Cambridge, MA, USA). 4′,6-diamidino-2-phenylindole (DAPI) and ProLong Gold were purchased from Invitrogen (Carlsbad, CA). An in situ cell death detection kit for terminal deoxynucleotidyl transferase dUTP nick end labeling (TUNEL) assay was purchased from Roche (In Situ Cell Death Detection Kit, Fluorescein; Roche Diagnostics, Indianapolis, IN).

### Culture of HCECs

A HCEC line was purchased from the American Type Culture Collection (ATCC, Manassas, VA). Cells were maintained in keratinocyte serum-free medium (GIBCO, Invitrogen, Carlsbad, CA) containing 5 ng/mL human recombinant epidermal growth factor, 0.05 mg/mL bovine pituitary extract, 0.005 mg/mL insulin, and 500 ng/mL hydrocortisone and were plated on dishes coated with 0.01 mg/mL fibronectin and 0.01 mg/mL bovine collagen type I. Cells were grown at 37°C in a humidified air atmosphere with 5% CO_2_, and the growth medium was changed daily. Subculturing was performed when the cell layers were confluent (2–3 days).

### Treatment of Drugs

The cells were divided into 2 groups (3 × 10^5^ cells per well in a 6-well culture plate, 6 × 10^3^ cells per well in a 96-well microtiter plate) and were allowed to attach for 24 hours; subsequently, the medium was removed. Cells were washed with Dulbecco phosphate-buffered saline, and then, the medium was replaced with drugs [0.0025%, 0.005%, and 0.01% BAC, preserved (BAC 0.0025%) 0.025% FML, preserved (0.005% BAC) 0.05% FML, preserved (0.01% BAC) 0.1% FML, and unpreserved (0.025%, 0.05%, and 0.1%) FML, 1% dimethyl sulfoxide (DMSO), original or diluted solvents, and unpreserved (0.025%, 0.05%, and 0.1%) FML with DMSO]. The cells were exposed to the drugs for 3, 5, and 10 minutes (5 minutes in the case of cell morphology analysis). The stimuli were removed after incubation. The cells were washed again with Dulbecco phosphate-buffered saline and then transferred to keratinocyte serum-free medium without drugs. The control solution was the same volume of phosphate-buffered saline (PBS); preserved and unpreserved FML were diluted with PBS to prepare formulations with different percentages of the drug.

### Analysis of Cell Morphology

Cells were incubated for an additional 6 hours to allow stabilization. Finally, the morphological features of cultured cells in 6-well plates were observed by phase-contrast microscopy. Images were captured at ×100 magnification.

### Cell Viability Assay

Cell Counting Kit-8 (CCK-8; Dojindo, Sunnyvale, CA) assay was used as a qualitative index of cell viability. Cells were plated in 96-well microtiter plates at a density of 6 × 10^3^ cells per well and were exposed to the drugs for 3, 5, and 10 minutes. Live cell count was then assayed using CCK-8 according to the manufacturer's protocol. Briefly, 10 μL of CCK-8 solution was added to each well, and the samples were incubated for 2 hours before the absorbance was measured at 450 nm. Data represent mean values obtained from 3 independent experiments.

### Animals and Procedures

Twenty 8 to 10-week-old female C57BL/6 mice (weight, 18–20 g; purchased from Hyochang Science, Daegu, South Korea) were used in this study. All experimental procedures were performed in accordance with the Association for Research in Vision and Ophthalmology Statement for the Use of Animals in Ophthalmic and Vision Research. On the basis of clinical evaluations (described below), the mice were randomly divided into 4 groups (5 mice in each group): normal saline- (control), 0.01% BAC-, preserved (0.01% BAC) 0.1% FML-, and unpreserved 0.1% FML-treated groups. For the first 2 weeks, dry eye syndrome was induced in the mice by topical administration of BAC.^26^ The right eyes of 15 randomly chosen mice were treated twice daily (9 am and 9 pm) topically with 5 μL of 0.2% BAC (BAC-treated group), whereas the other 5 mice were treated with normal saline in the right eye (control group). BAC powder was dissolved in normal saline to prepare 0.2% BAC solution. Over the next 2 weeks, the 15 mice treated with 0.2% BAC were randomly divided into 3 groups. Five microliters of drugs [0.01% BAC (n = 5), preserved (0.01% BAC) 0.1% FML (n = 5), and unpreserved 0.1% FML (n = 5)] were administered to the mice in the 3 groups. The mice in the control group (n = 5) were administered 5 μL of normal saline for 2 weeks. The right eyes of all the mice were treated thrice daily (9 am, 3 pm, and 9 pm) with topical drops. During the treatment, clinical evaluations were performed by a single masked ophthalmologist. On day 30, all the mice were killed. Eye tissues were harvested carefully for histological analysis after the methods described below.

### Paraffin Embedding and Histological Examination

Corneal tissues were isolated from the harvested eye tissues. The collected corneal tissues were fixed in 4% paraformaldehyde in PBS for 24 hours at 4°C and then washed twice in PBS. The samples were then dehydrated through an ethanol series, cleared by soaking in xylene, embedded in paraffin, and sectioned into 5-μm slices using a microtome, RM 2125RT (Leica, Wetzlar, Germany). Slides containing paraffin sections were deparaffinized in xylene and rehydrated through an ethanol series, and then, hematoxylin-eosin (H&E) staining was performed for histopathological examination. The specimens were mounted with Permount (Fisher, Fair Lawn, NJ). Images were captured using an Axio Vision 4 microscope (Carl Zeiss, Jena, Germany).

### TUNEL Assay

The specimens were subjected to TUNEL assay using an in situ cell death detection kit, fluorescein (Roche Diagnostics, Cat. No. 11 684 795 910), in accordance with the manufacturer's instructions. The positive control was incubated with deoxyribonuclease I (3000 U/mL in 50 mM Tris/HCl), and the negative-control sample was incubated with label-solution only. TUNEL-positive cells were viewed by Zeiss fluorescence microscope (Axio Vision 4; Carl Zeiss). The total number of cells and number of TUNEL-positive cells were counted in 5 individual high-power fields in each of the 5 samples. All slides were read by an experienced scientist who was masked to the evaluation, and the scores and average ratio of the number of TUNEL-positive cells to the total number of cells were calculated.

### Immunofluorescence Staining

For immunofluorescence staining, sectioned corneal tissue slides were deparaffinized in xylene and then rehydrated through an ethanol series. The specimens were permeabilized and blocked overnight with 5% normal goat serum and bovine serum albumin in TBS. The specimens were then incubated with monoclonal or polyclonal antibodies against TNF-α, IL-6, and HLA-DR (diluted 1:50–100) and were further incubated with the appropriate Alexa Fluor 488- or 555-conjugated secondary antibody at room temperature for 1 hour, and the nuclei were counterstained with DAPI (1 μg/mL). The slides were mounted with ProLong Gold, and images were captured using a Zeiss fluorescence microscope (Axio Vision 4; Carl Zeiss). Density of green or red fluorescence was measured in color histogram using the ImageJ software program.

### Statistical Analysis

Each experiment was repeated 3 or more times. Results are presented as means ±SD. Differences between various data sets were tested for significance using a Student *t* test or analysis of variance, and *P* values less than 0.05 were considered statistically significant. An *F*-test was performed to ensure homogeneity of variances. The independent-samples *t* test was used to compare the means of 2 independent samples (control group versus treatment group or between the preserved- and unpreserved-FML groups at the same time point and dose). If the *P*-value was high (*P* > 0.05), a *t* test assuming equal variance was performed; otherwise, a *t* test assuming unequal variance was performed.

## RESULTS

### Cytotoxic Effect of BAC and Preserved FML in Cultured HCECs In Vitro

**FIGURE 1:**
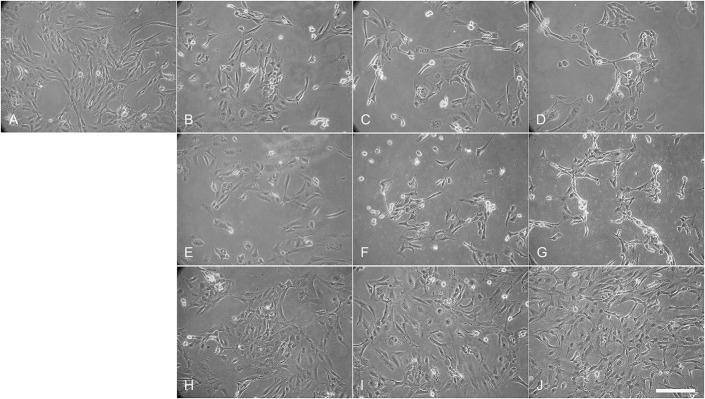
BAC-induced morphological changes and cell death in immortalized HCECs. The morphological features of HCECs at different concentrations of drugs were examined in 6-well plates by phase-contrast microscopy (original magnification, ×100). All experiments were performed in triplicate. A, PBS control, (B–D) 0.0025%, 0.005%, and 0.01% BAC, (E–G) preserved (0.0025% BAC) 0.025% FML, preserved (0.005% BAC) 0.05% FML, and preserved (0.01% BAC) 0.1% FML, and (H–J) unpreserved 0.025%, 0.05%, and 0.1% FML. Scale bar, 200 μm.

To investigate the cytotoxicity of the drugs at different concentrations in HCECs, the cells were exposed to the drugs for 5 minutes after removing the stimuli and were incubated for a further 6 hours. We observed morphological changes by phase-contrast microscopy (magnification, ×100). BAC and BAC-containing FML caused apoptotic changes, such as cell shrinkage, appearance of an apoptotic body, and detachment from the plate, in a dose-dependent manner (Fig. 1). Under the control condition, cells did not show any obvious apoptosis (Fig. 1A). By contrast, after treatment with 0.0025% BAC (B) and preserved 0.025% FML (E), some cells were detached and washed out, respectively. After treatment with 0.005% (C) and 0.01% BAC (D), and 0.05% (F) and 0.1% (G) preserved FML, respectively, many cells presented shrunken cytoplasm and intact plasma membrane, which is indicative of apoptosis. Importantly, it seems likely that cultured cells were not very sensitive to unpreserved-FML treatments (Figs. 1H–J).

### Differential Cell Viability on BAC, and Preserved- or Unpreserved-FML Treatments in a Dose- and Time-Dependent Manners in HCECs In Vitro

**FIGURE 2:**
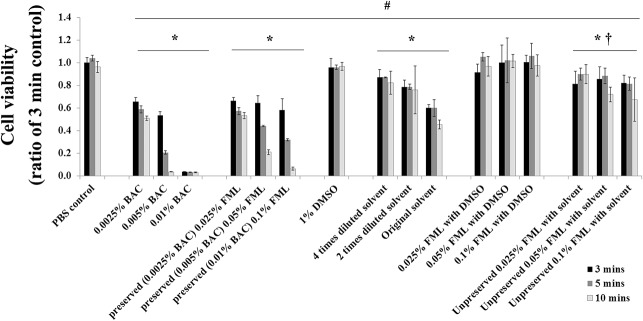
Alteration of BAC-induced cell cytotoxicity in immortalized HCECs. Cell cytotoxicity and viability were measured using CCK-8. The results are presented as mean ± SD of 5 independent experiments (n = 5), each conducted in triplicate. *P < 0.005 versus each 3-minute control, as deduced from an independent *t* test, †*P* < 0.001 versus solvent alone, as deduced from an independent *t* test, and #*P* < 0.05, as deduced by 1-way analysis of variance.

To investigate whether BAC or preserved (BAC) FML formulations reduce cell viability, the cells were exposed to the drugs for 3, 5, and 10 minutes, and then cell viability was analyzed by CCK-8 assay. The results revealed that the cell viability of HCECs decreased on exposure to BAC or preserved (BAC) FML. The CCK-8 assay demonstrated a dose- and time-dependent toxic effect with increasing concentrations of BAC or preserved (BAC) FML in HCECs (Fig. 2). At all doses and time points, significant toxicity was observed in BAC [cell death rates: 3 minutes, 34.5% ± 3.8, 46.7% ± 3.3, and 96.4% ± 0.2 (*P* < 0.001); 5 minutes, 41.3% ± 1.3, 79.4% ± 1.15, and 96.8% ± 1.1 (*P* < 0.005); 10 minutes, 49.2% ± 2.11, 96.4% ± 0.3, and 96.8% ± 1.4 (*P* < 0.001) for 0.0025%, 0.005%, and 0.01% BAC, respectively] or preserved FML [3 minutes, 33.6% ± 2.7, 35.8% ± 6.4, and 41.91% ± 0.99 (*P* < 0.001); 5 minutes, 42.9% ± 3.0, 55.9% ± 0.6, and 68.1% ± 0.9 (*P* < 0.001); 10 minutes, 46.7% ± 2.7, 79.1% ± 2.1, and 93.6% ± 1.2 (*P* < 0.001) for 0.0025%, 0.005%, and 0.01% BAC-containing FML, respectively]-treated HCECs. However, this cytotoxic effect was somewhat overcome by preserved FML treatment. Compared with preserved FML, cell death was decreased significantly on treatment with unpreserved FML with original solvent [3 minutes, 18.7% ± 11.2, 14.7% ± 11.1, and 18.1% ± 6.9 (*P* < 0.01); 5 minutes, 10.1% ± 5.4, 11.7% ± 6.8, and 18.6% ± 5.7 (*P* < 0.02); and 10 minutes, 10.2% ± 8.5, 28.1% ± 6.3, and 32.8% ± 19.3 (*P* < 0.001) for 0.025%, 0.05%, and 0.1% FML, respectively]. It is interesting to note that original solvent used for dissolving FML caused significant toxicity compared with PBS control [3 minutes, 13.0% ± 7.0, 21.4% ± 6.1, and 40.1% ± 3.0 (*P* < 0.01); 5 minutes, 13.0% ± 0.4, 21.4% ± 6.8, and 40.1% ± 7.4 (*P* < 0.02); and 10 minutes, 17.8% ± 8.6, 24.1% ± 21.3, and 54.7% ± 3.8 (*P* < 0.001) for 4 times, 2 times diluted, and original solvent, respectively] in dose- and time-dependent manner. Based on these results, it seems likely that the cytotoxic effect of original solvent alone was overcome by FML, suggesting that FML itself may not cause toxicity. Interestingly, a recent study showed that migration of Caki-1 cells, a renal clear cell carcinoma cell line, treated with FML was reduced compared with DMSO-treated control.^27^ Based on a previous study, we also examined whether FML dissolved in DMSO affects cytotoxicity in cultured HCECs in vitro. As expected, similar to PBS and DMSO controls, we observed that there was a weak cytotoxic effect of unpreserved FML (0.025%, 0.05%, and 0.1%) dissolved in DMSO in cultured HCECs in vitro at all the time points. Taken together, our result indicates that unpreserved FML has no cytotoxic effect and suggests the potential usefulness of topical unpreserved FML without use of BAC which induced ocular surface diseases.

### Histological Analysis by H&E Staining After Treatment of Topical Eye Drops Containing Unpreserved or Preserved FML in BAC-Induced Dry Eye in Mice In Vivo

**FIGURE 3:**
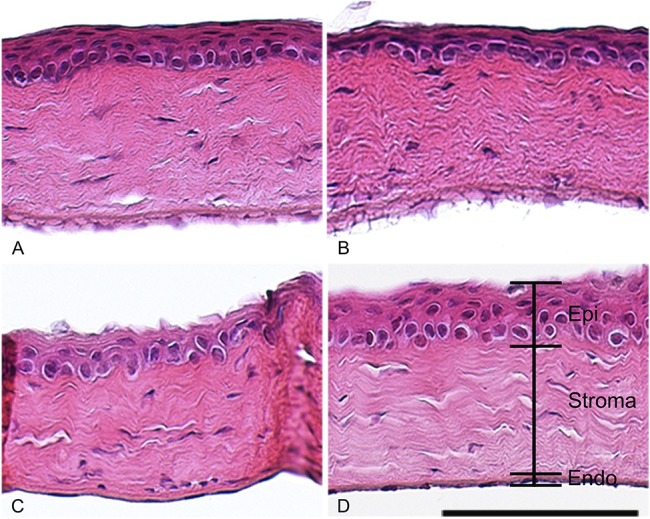
Corneal histological changes observed after BAC and/or FML treatment in the BAC-induced dry eye C57BL/6 mouse model. The cellular distribution and overall structure of the cornea were examined by H&E staining. A, Normal saline, (B) 0.01% BAC, (C) preserved (0.01%) 0.1% FML, and (D) unpreserved 0.1% FML. Cells from the unpreserved FML (D) and control groups (A) had similar morphologies. Cells from the 0.01% BAC- (B) and preserved (0.01%) 0.1% FML-treated (C) groups exhibited surface desquamation, irregular surface, loss of cell borders, and stromal shrinkage. These experiments were performed in triplicate. Scale bar, 100 μm. Endo, endothelium; Epi, epithelium; Stroma, corneal stroma.

To test the possibility of corneal damage due to BAC, we performed immunohistochemistry by H&E staining (Fig. 3). Morphological changes were observed in sectioned corneal tissues treated with normal saline, 0.1% BAC, preserved (0.01% BAC) 0.1% FML, and unpreserved 0.1% FML. No apparent structural abnormalities were detected in mice corneas subjected to normal saline treatment (Fig. 3A). However, mice corneas exposed to 0.1% BAC showed severe structural damage, including surface desquamation, irregular surface, cell border loss, anisocytosis, and stromal shrinkage, after induction of dry eye syndrome by treatment with 0.2% BAC for 2 weeks (Fig. 3B). Interestingly, mice treated with preserved FML still exhibited some signs of dry eye syndrome on the superficial corneal surface (Fig. 3C). As expected, unpreserved FML almost reversed dry eye syndrome in the corneal surface, and the corneal surface of unpreserved FML-treated mice was similar to that of the control group mice (Fig. 3D). These data suggest the potential usefulness of topical unpreserved FML without BAC for the clinical treatment of ocular surface disease which may associate with dry eye.

### TUNEL Staining After Treatment of Topical Eye Drops Containing Unpreserved or Preserved FML in BAC-Induced Dry Eye in Mice In Vivo

**FIGURE 4:**
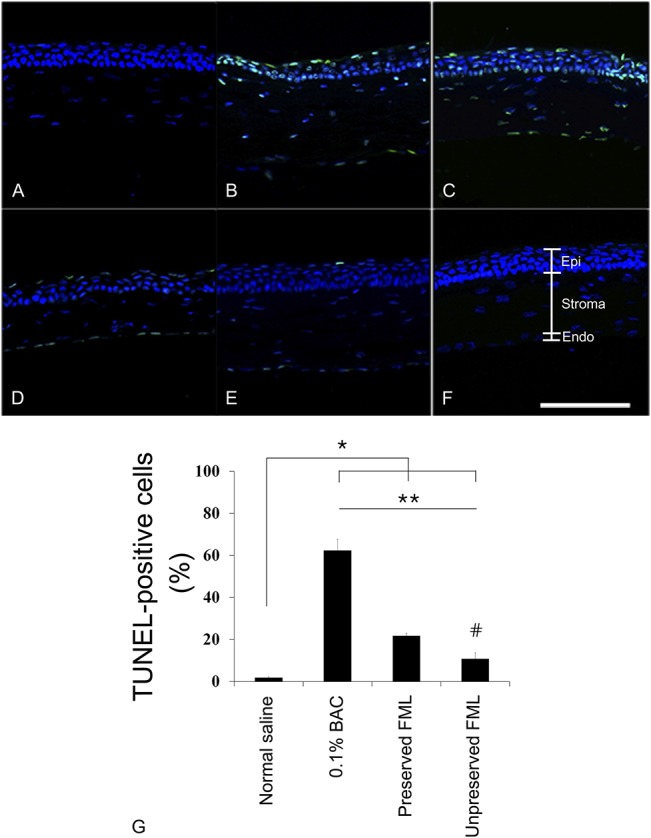
Representative image showing the distribution of apoptotic cells in corneal tissue treated with BAC and/or FML for 2 weeks. TUNEL assay were to detect apoptotic nuclei. A, Normal saline, (B) 0.01% BAC, (D) preserved (0.01% BAC) 0.1% FML, (E) unpreserved 0.1% FML, (C) positive control, and (F) negative control. TUNEL staining revealed apoptotic cells in BAC. FML treatment significantly reduced the number of apoptotic cells. G, Mean superficial apoptotic cells are shown. All experiments were performed in triplicate. The results in the bar graph are the average of 3 independent experiments. *P < 0.05 versus normal saline control, as deduced from an independent *t* test, **P < 0.005, as deduced by 1-way analysis of variance, and #*P* < 0.01 versus preserved FML, as deduced from an independent *t* test. Scale bar, 100 μm. Endo, endothelium; Epi, epithelium; Stroma, corneal stroma.

To detect apoptosis of corneal tissue induced by the drugs, we performed a TUNEL assay (Fig. 4). Similar to positive control (Fig. 4C), BAC dramatically induced apoptosis in the corneal epithelium, stroma, and endothelium (Fig. 4B). However, preserved FML-treated corneas showed few apoptotic cells in the superficial corneal epithelium and endothelium (Fig. 4D). Interestingly, cell death response was markedly decreased in the corneas of the FML-treated group, compared with the BAC-treated group. Surprisingly, a few TUNEL-positive cells were also observed in unpreserved FML-treated corneas, but were less than preserved FML (Fig. 4E). Similar to control by normal saline (Fig. 4A), there was no TUNEL staining in negative control (Fig. 4F). Statistically, as shown in Figure 4G, treatment with BAC markedly increased the number of TUNEL-positive cells (62.3% ± 5.4, *P* < 0.05), whereas preserved and unpreserved FML-treated corneas showed dramatically reduced numbers of TUNEL-positive cells (21.8% ± 1.3 and 10.8% ± 2.8, respectively, *P* < 0.05). These results suggest that unpreserved FML was more effective for cell survival than preserved FML.

### Differential Distribution Patterns of TNF-α, IL-6, and HLA-DR After Exposure of Drugs in Dry Eye-Induced Mice

**FIGURE 5:**
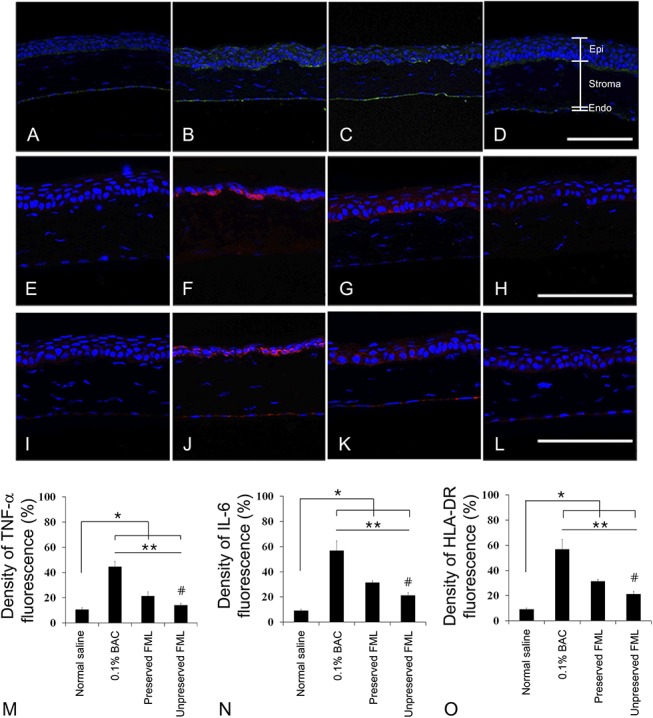
Differential expression of TNF-α, IL-6, and HLA-DR in BAC-induced dry eye C57BL/6 mouse model. Immunofluorescence staining was performed with TNF-α-specific antibody (green; A–D), IL-6-specific antibody (red; E–H), and HLA-DR-specific antibody (red; I–L) in corneal tissues. (A, E, and I) Normal saline, (B, F, and J) 0.01% BAC, (C, G, and K) preserved (0.01% BAC) 0.1% FML, and (D, H and L) unpreserved 0.1% FML. (M–O) To quantify the results, bar charts for the relevant images are shown. Quantification of green- or red-fluorescence density was performed using the ImageJ software program. These experiments were performed in triplicate, and the results are the average of 3 independent experiments. The results of the bar graph are the averages of 3 independent experiments. *P < 0.01 versus normal saline control, as deduced from an independent *t* test, **P < 0.005, as deduced by 1-way analysis of variance, and #*P* < 0.05 versus preserved FML, as deduced from an independent *t* test. Scale bar, 100 μm. Endo, endothelium; Epi, epithelium; Stroma, corneal stroma.

To determine the distribution and expression patterns of inflammatory marker proteins after exposure to drugs, we performed immunofluorescence staining with specific antibodies against TNF-α, IL-6, and HLA-DR (TNF-α; Figs. 5A–D, IL-6; Figs. 5E–H, and HLA-DR; Figs. 5I–L). As expected, strong positive signals for TNF-α (Fig. 5B) and HLA-DR (Fig. 5J) in both corneal epithelium and endothelium, and for IL-6 in the epithelium (Fig. 5F) were detected in the BAC-treated groups. Compared with BAC-treated mice, staining density of all marker proteins was strongly decreased in the corneas of preserved FML-treated mice (TNF-α; Fig. 5C, IL-6; Fig. 5G, and HLA-DR; Fig. 5K). Most importantly, expression of TNF-α (Fig. 5D), IL-6 (Fig. 5H), and HLA-DR (Fig. 5L) in unpreserved FML-treated groups was nearly undetected in the corneal epithelium and endothelium at all. To quantify these results, bar charts for the relevant images have been included (Figs. 5M–O). Quantification of the fluorescence intensity demonstrated that the expression of BAC-induced inflammatory marker proteins was dramatically increased in BAC-treated mice (Fig. 5B; 44.6% ± 4.0, Fig. 5F; 56.9% ± 7.6, and Fig. 5J; 56.6% ± 7.9, respectively, *P* < 0.05). However, unpreserved FML-treated corneas exhibited markedly reduced expression of inflammatory proteins. Interestingly, unpreserved FML (Fig. 5D; 14.1% ± 1.3, Fig. 5H; 21.4% ± 2.1, and Fig. 5L; 14.4% ± 1.9, respectively, *P* < 0.05) was over 1.5-fold more effective than preserved FML in reversing dry eye syndrome (Fig. 5C; 21.3% ± 3.3, Fig. 5G; 31.4% ± 1.5, and Fig. 5K; 23.4% ± 0.1, respectively, *P* < 0.05). Level of TNF-α, IL-6, and HLA-DR fluorescence intensity in normal saline groups were detected in 10% or less. Taken together, these data suggest that rapid antiinflammatory effect with unpreserved FML rather than preserved FML is very effective for mice with moderate dry eye.

## DISCUSSION

The preservatives used in most eye drops provide antimicrobial activity in multiuse bottles, restricting secondary bacterial, mycotic, and amoebal ocular infection caused by contaminated solutions, and prolonging the shelf life of the drug by inhibiting biodegradation and maintaining drug efficacy.^28^ Cytotoxicity in the ocular surface cells is a well-known harmful effect of BAC-containing topical eye drop solutions. In our previous study, we demonstrated the toxicity of BAC-preserved, polyquad-preserved, and preservative-free prostaglandin analogs on primary cultured human conjunctival fibroblast cells.^29^ Many recent studies have reported extensive detachment of cells from the culture dish, increased secretion of proinflammatory cytokines, and cell death in response to BAC-containing solutions.^30^–^32^ In this study, we investigated cell apoptosis and cytotoxicity by BAC treatment in HCECs (Figs. 1 and 2). Our results revealed that BAC and preserved (BAC) FML induced cell cytotoxicity, as evidenced by altered cell morphology and the results of the CCK-8 assay in primary cultured HCECs. BAC exposure induced cell cytotoxicity even at low doses (Fig. 1B), suggesting that even dilute BAC formulations can damage the ocular surface. Cell detachment (Fig. 1D) and wash-out (nearly 100% effect) were observed on exposure to undiluted BAC (0.01% BAC) for 10 minutes (Fig. 2). Although cytotoxicity was detected slightly in unpreserved FML with solvent, because FML with DMSO has no cytotoxicity (Fig. 2), it can be considered to be the solvent effect. BAC induced cytotoxicity not only in corneal epithelial cells but also in conjunctival cells (data not shown).

Dry eye disease is a common multifactorial ocular surface disease.^19^ A stable rabbit dry eye model was established by topical application of 0.1% BAC for 5 weeks; after BAC removal, symptoms of dry eye syndrome sustained for 2 to 3 weeks.^33^ Topical administration of 0.2% BAC for 1 week in mice successfully induced dry eye condition.^26^ Therefore, we applied BAC to induce dry eye disorder by causing inflammatory damage to the corneal and conjunctival epithelial cells. This mouse model was appropriate for the study of inflammatory dry eye. In our mouse model, we observed surface desquamation, irregular surface, loss of cell borders, and stromal shrinkage (Fig. 3B). Increased epithelial apoptotic rate was observed in the TUNEL assay (Fig. 4B) on treatment with 0.01% BAC. Topical steroids, through several mechanisms of action, help reduce ocular inflammation.^34^–^36^ Although preserved (BAC) FML contained the same concentration of BAC, the antiinflammatory effect of FML led to reduced apoptosis in corneal tissue (Fig. 4D).

BAC is a component of all multidose eye drop formulas, and it can induce corneal epithelial dysfunction, which can damage the corneal epithelial barrier even at very low doses.^9^ BAC is degraded into hydrogen peroxide, which can cause oxidative stress and trigger inflammatory responses in corneal and conjunctival epithelial cells.^37^ Even at low concentrations, BAC induces a marked increase in the expression levels of several inflammatory cytokines, such as IL-1, IL-6, IL-10, IL-12, TNF-α, and HLA-DR in HCECs.^38^,^39^ Hyperosmolarity of tears, caused by BAC-induced dry eye, can stimulate the production of inflammatory cytokines.^19^,^20^ Our results revealed that BAC increases the expression of inflammatory marker proteins, such as TNF-α (44.6% ± 4.0, *P* < 0.05), IL-6 (56.9% ± 7.6, *P* < 0.01), and HLA-DR (56.6% ± 7.9, *P* < 0.02; Figs. 5B, G, and L) when compared with normal saline. Inflammation is the key characteristic of ocular surface disease, such as dry eye. Corticosteroids are commonly used in antiinflammatory eye drops, such as FML to control eye inflammation.^25^,^40^ In a recent study, topical application of unpreserved 0.1% FML was shown to be an effective and relatively safe treatment for patients with dry eye syndrome.^41^ This clinical result suggests that unpreserved 0.1% FML replicates the effects of corticosteroids, has no side effects from preservatives, and is highly effective and safe for the treatment of inflammatory dry eye syndrome. In our study, cells were not sensitive to unpreserved FML exposure. Although unpreserved 0.1% FML-treated cells exhibited mild cell shrinkage and detachment from plates, almost all cells were stable on the plate, and cell morphology was normal (Figs. 1H–J), when compared with cells treated with preservative-containing FML (Figs. 1E–G). In the cell viability assay (Fig. 2), compared with BAC-only solution, preservative-containing FML afforded significant protective effects (11%–24% in 5 minutes and 4%–55% in 10-minute exposure time). No significant differences were observed between cells treated with 0.025% FML with 0.0025% BAC and those treated with 0.0025% BAC alone. In the case of unpreserved FML, cell toxicity was negligible, and cell viability was over 2-fold higher than that observed for preserved FML at all the time points and doses. We confirmed the therapeutic effect and safety of unpreserved FML by H&E staining (Fig. 3). The percentage of TUNEL-positive cells was 10.8% ± 2.8 for unpreserved FML and 21.8% ± 1.3 for preserved FML, suggesting that unpreserved FML is twice as effective as preserved FML in reducing surface damage (Figs. 4D, E, and G).

Corticosteroids are strong inhibitors of inflammation and have been proven to decrease the production of Il-1α, Il-1β, and TNF-α by inhibiting the function of mitogen-activated protein kinases.^42^ Similarly, in this study, preserved FML decreased TNF-α, IL-6, and HLA-DR expression in BAC-induced dry eye mouse cornea (Figs. 5C, G, and K). Moreover, therapeutic effect against BAC-induced inflammation was highest for unpreserved FML (Figs. 5D, H, and L).

In summary, this study confirms that topical application of the corticosteroid, FML, suppress the expression of inflammatory cytokines, but BAC, which is present in FML formulations, has cytotoxic effects on ocular surface cells and tissues, and therefore, decreases the efficacy of FML treatment. Accordingly, unpreserved FML is more effective than preserved eye drops in decreasing ocular inflammation and improving dry eye symptoms. However, further studies are necessary to clarify the precise intracellular signal mechanism underlying BAC-induced cytotoxicity in FML treatment.
